# Biologic Therapy Is Associated with Reduced Pleural-Line Thickness in Severe Asthma: A Prospective Transthoracic Ultrasound Study

**DOI:** 10.3390/jcm15166346

**Published:** 2026-08-17

**Authors:** Müge Erbay, Selçuk Akkaya, Funda Öztuna, Hatice Tayar

**Affiliations:** 1 Section of Immunology and Allergy Diseases, Department of Chest Diseases, Medical Faculty, Karadeniz Technical University, 61080 Trabzon, Turkey; 2 Department of Radiology, Medical Faculty, Karadeniz Technical University, 61080 Trabzon, Turkey; selcuk.akkaya85@gmail.com (S.A.); tayar.hatice61@gmail.com (H.T.); 3 Department of Chest Diseases, Medical Faculty, Karadeniz Technical University, 61080 Trabzon, Turkey; foztuna@yahoo.com

**Keywords:** severe asthma, biologic therapy, lung ultrasound, pleural-line thickness, small-airway disease, treatment response

## Abstract

**Background/Objectives:** Tissue-level markers of treatment response are lacking in severe asthma. Transthoracic ultrasound (TUS) non-invasively detects pleural-line and subpleural abnormalities that have been linked to small-airway disease, but it has not been evaluated as a marker of response to biologic therapy. The primary objective was to determine whether mean pleural-line thickness in the right and left hemithoraces changed between baseline and week 16 of biologic therapy. The secondary objectives were to describe the accompanying changes in pleural-line regularity, lung function, symptom control, and quality of life, and to examine whether the ultrasound changes are related to the clinical and functional response. **Methods:** In this prospective, single-center study, 41 adults with severe asthma who were starting omalizumab (*n* = 18), mepolizumab (*n* = 12), or benralizumab (*n* = 11) underwent standardized 14-zone TUS, spirometry, the Asthma Control Test (ACT), and the Asthma Quality of Life Questionnaire (AQLQ) at baseline and after 16 weeks. Pleural-line thickness and pleural-line regularity score were the primary TUS measures. Paired and between-group tests and Spearman rank correlations were used. **Results:** At week 16, 20 patients (48.8%) were complete responders. The mean ACT score increased from 13.2 ± 4.1 to 22.6 ± 2.9, the mean AQLQ total score from 3.6 ± 1.2 to 4.9 ± 1.2, FEV1 from 86.0 ± 24.8% to 99.0 ± 20.9% predicted, and FEF25–75 from 61.4 ± 35.0% to 81.2 ± 33.4% predicted (all *p* ≤ 0.001), whereas the blood eosinophil count fell from a median of 400 (0–1990) to 80 (0–330) cells/µL (*p* < 0.001). Median pleural-line thickness decreased from 1.27 to 0.94 mm in the right hemithorax and from 1.31 to 0.97 mm in the left hemithorax (both *p* < 0.001), and the pleural-line regularity score increased on both sides (both *p* < 0.001). Pleural-line thickness decreased significantly in all three biologic groups (all *p* ≤ 0.001), with no evidence of a differential treatment effect. Changes in pleural-line thickness did not correlate with ΔACT, ΔAQLQ, ΔFEV1, or ΔFEF25–75 (all *p* > 0.05). **Conclusions:** After 16 weeks of biologic therapy, pleural-line thickness was reduced and the pleural-line regularity score had improved on TUS. Because these pleural changes did not correlate with the symptomatic or spirometric response, TUS may capture a complementary, tissue-level dimension of the treatment effect. As the study was an uncontrolled single-arm design, the changes cannot be attributed to the biologic agent itself, and prospective controlled validation is required before TUS can be proposed as a marker of response.

## 1. Introduction

Asthma is a heterogeneous chronic inflammatory airway disease that affects more than 300 million people worldwide [1]. Approximately 5–10% of patients have severe asthma, which, despite optimized inhaled therapy, is associated with frequent exacerbations, impaired quality of life, accelerated decline in lung function, and increased healthcare utilization [2]. Biologic therapies directed against type 2 inflammatory pathways have substantially improved outcomes in severe asthma by reducing exacerbations, improving symptom control, and lowering oral corticosteroid exposure. Response nevertheless varies considerably between individuals, so that accurate assessment of the effectiveness of a biologic agent in a given patient remains an important clinical challenge.

Current guidelines recommend a multidimensional assessment of biologic response that combines symptom control, exacerbation frequency, lung function, oral corticosteroid use, biomarkers such as the blood eosinophil count and fractional exhaled nitric oxide (FeNO), and patient-reported outcomes [1]. No single parameter, however, reliably reflects the underlying biological response to treatment.

Clinical improvement frequently exceeds spirometric improvement, and peripheral blood eosinophils do not necessarily represent mirror ongoing tissue inflammation [3]. FeNO, sputum eosinophil counts, and advanced imaging provide additional information, but their routine use is constrained by availability, cost, technical complexity, or invasiveness. An accessible, objective marker capable of reflecting tissue-level change during biologic therapy is therefore still lacking.

Small-airway dysfunction is increasingly recognized as a major contributor to asthma severity, persistent symptoms, airflow limitation, and airway remodeling [4]. Structural change in the distal lung is conventionally assessed with high-resolution computed tomography (HRCT) or magnetic resonance imaging, but these techniques are expensive, HRCT exposes patients to ionizing radiation, and neither is well suited to repeated longitudinal assessment [5]. Interest has consequently grown in non-invasive imaging methods that allow peripheral airway abnormalities to be evaluated repeatedly during routine follow-up.

Transthoracic ultrasonography (TUS) has emerged as a rapid, bedside, radiation-free imaging modality that is now widely used in respiratory medicine [6]. Normally aerated lung cannot be visualized directly, but abnormalities of the pleura and subpleural lung are readily assessable. Recent studies suggest that pleural-line thickening, a lower pleural-line regularity score, and abnormal pleural sliding are associated with air trapping and small-airway dysfunction in severe asthma, and show good agreement with HRCT findings [5,7]. In parallel, CT, MRI, oscillometry, and histopathological studies have shown that biologic therapies reduce airway remodeling and improve distal airway abnormalities [8,9,10]. Whether such structural improvement can also be detected with TUS is unknown.

Although pleural-line abnormalities have been described on transthoracic ultrasonography in patients with severe asthma, it remains unclear whether they represent stable markers of disease severity or dynamic features that change with treatment. The potential role of TUS as a longitudinal, non-invasive imaging biomarker for monitoring response to biologic therapy has therefore not been established. This prospective study was designed to determine whether pleural-line parameters change after 16 weeks of biologic therapy and whether any such changes are associated with established clinical and functional measures of treatment response.

## 2. Materials and Methods

### 2.1. Study Design

This prospective, single-center study was conducted between June 2024 and January 2026 as a collaboration between the Departments of Chest Diseases, Immunology and Allergy, and Radiology at Karadeniz Technical University Faculty of Medicine.

Inclusion criteria were as follows:
  - age ≥ 18 years;
  - a diagnosis of severe asthma according to Global Initiative for Asthma (GINA) criteria [1];
  - initiation of biologic therapy during the study period;
  - written informed consent;
  - availability of both baseline and week-16 transthoracic ultrasonography (required for inclusion in the longitudinal TUS analyses).
Exclusion criteria were as follows:
  - no indication for biologic therapy;
  - refusal to participate;
  - no follow-up transthoracic ultrasonography;
  - interstitial lung disease, pleural disease, or an active respiratory infection within the preceding 4 weeks.

The biologic agent was selected by the treating physician in accordance with GINA recommendations, taking into account the eosinophil count, total IgE level, allergen sensitization, exacerbation history, and comorbidities. Patients who had no indication for biologic therapy and those who declined to participate were excluded. Only patients with both baseline and follow-up transthoracic ultrasonography were included in the longitudinal TUS analyses.

The study was approved by the Karadeniz Technical University Ethics Committee (Approval No: 2023/211) and supported by the Karadeniz Technical University Scientific Research Projects Coordination Unit (Project No: 11138).

Generative artificial intelligence was used only to assist in preparing the graphical abstract and for English-language editing. It was not used for study design, data collection, analysis, interpretation, or the generation of scientific conclusions. The authors reviewed and approved all final content and take full responsibility for the manuscript.

### 2.2. Clinical Characteristics

Demographic characteristics, including age, sex, smoking status, and body mass index (BMI), were recorded, together with comorbidities and asthma duration. Pulmonary function test (PFT) parameters (FEV1/FVC, FEV1, FVC and FEF25–75), current treatments (montelukast, long-acting muscarinic antagonists and biologic agents), asthma-related emergency visits, systemic corticosteroid use, and hospitalization history were obtained from the patient records. Laboratory data comprised total serum IgE levels and peripheral blood eosinophil counts.

### 2.3. Clinical Assessments

Asthma symptom control was assessed with the Asthma Control Test (ACT) at baseline and 16 weeks after the initiation of biologic therapy. ACT scores were categorized as uncontrolled (5–15), partially controlled (16–19), or well controlled (20–25).

Health-related quality of life was assessed with the validated Turkish version of the Asthma Quality of Life Questionnaire (AQLQ) at the same two time points [11]. The AQLQ comprises 32 items in four domains: symptoms (12 items), activity limitation (11 items), emotional function (5 items), and environmental stimuli (4 items). Each item is scored on a 7-point scale (1 = maximum impairment, 7 = no impairment). Domain scores and the total score were calculated separately, with higher scores indicating better quality of life.

FEF25–75 (% predicted) was evaluated as an exploratory spirometric marker of small-airway function.

### 2.4. Assessment of Treatment Response

Treatment response was assessed at week 16 and classified according to the predefined criteria summarized in Table 1. The classification was defined a priori by the investigators on the basis of clinically meaningful thresholds for asthma control, lung function, and exacerbation outcomes reported in previous severe asthma studies, rather than by applying a previously validated composite response score [12].

### 2.5. Transthoracic Ultrasonography

All patients underwent TUS at baseline and 16 weeks after initiation of biologic therapy. To minimize confounding, patients were asked to withhold long-acting muscarinic antagonists for 24 h and long-acting β2-agonists for 12 h before imaging, and to avoid smoking for 4 h and strenuous physical activity before the examination.

Examinations were performed with the patient seated, using a GE Voluson E10 system (GE Healthcare, Chicago, IL, USA) with a linear transducer (3.33–10 MHz) and a dedicated lung preset, at a depth of 5 cm, a gain of 60 dB, and the focal zone positioned at the pleural line.

All examinations were performed by a single experienced thoracic radiologist who was blinded to the patients’ clinical characteristics, pulmonary function results, symptom and quality-of-life scores, and treatment-response classification; none of these data were available at the time of image acquisition or measurement. The radiologist was aware that the patients were receiving treatment for severe asthma and of the visit order, but no expected direction or magnitude of change in the ultrasound parameters was specified. To minimize expectation and measurement bias, examinations followed a standardized 14-zone protocol with predefined anatomical landmarks and caliper-placement rules. Baseline measurements were not consulted during the week-16 examination, and images were not re-measured after treatment-response classification became available. The same ultrasound system and identical acquisition settings, including preset, transmit frequency, depth, gain, and focal-zone placement, were used for each patient at both examinations.

Imaging was performed bilaterally along the parasternal, midclavicular, anterior axillary, midaxillary, posterior axillary, midscapular, and paravertebral lines, giving 14 lung zones (7 per hemithorax). The posterior zones were obtained with the patient seated and the arms brought forward to open the scapular and paravertebral windows; the small footprint of the linear transducer allowed adequate visualization of the pleural line through the narrow intercostal spaces in these positions. All 14 zones were adequately visualized in every patient at both examinations; no zone was non-diagnostic and no measurement was missing. A total of 1148 pleural-line thickness measurements and 1148 morphological assessments were therefore obtained (41 patients × 14 zones × 2 examinations).

The following parameters were recorded in each zone: pleural sliding (gliding sign), pleural-line thickness (the maximum pleural-line thickness in the zone), pleural-line morphology (regular or irregular), and the presence of subpleural nodules.

**Figure 1:**
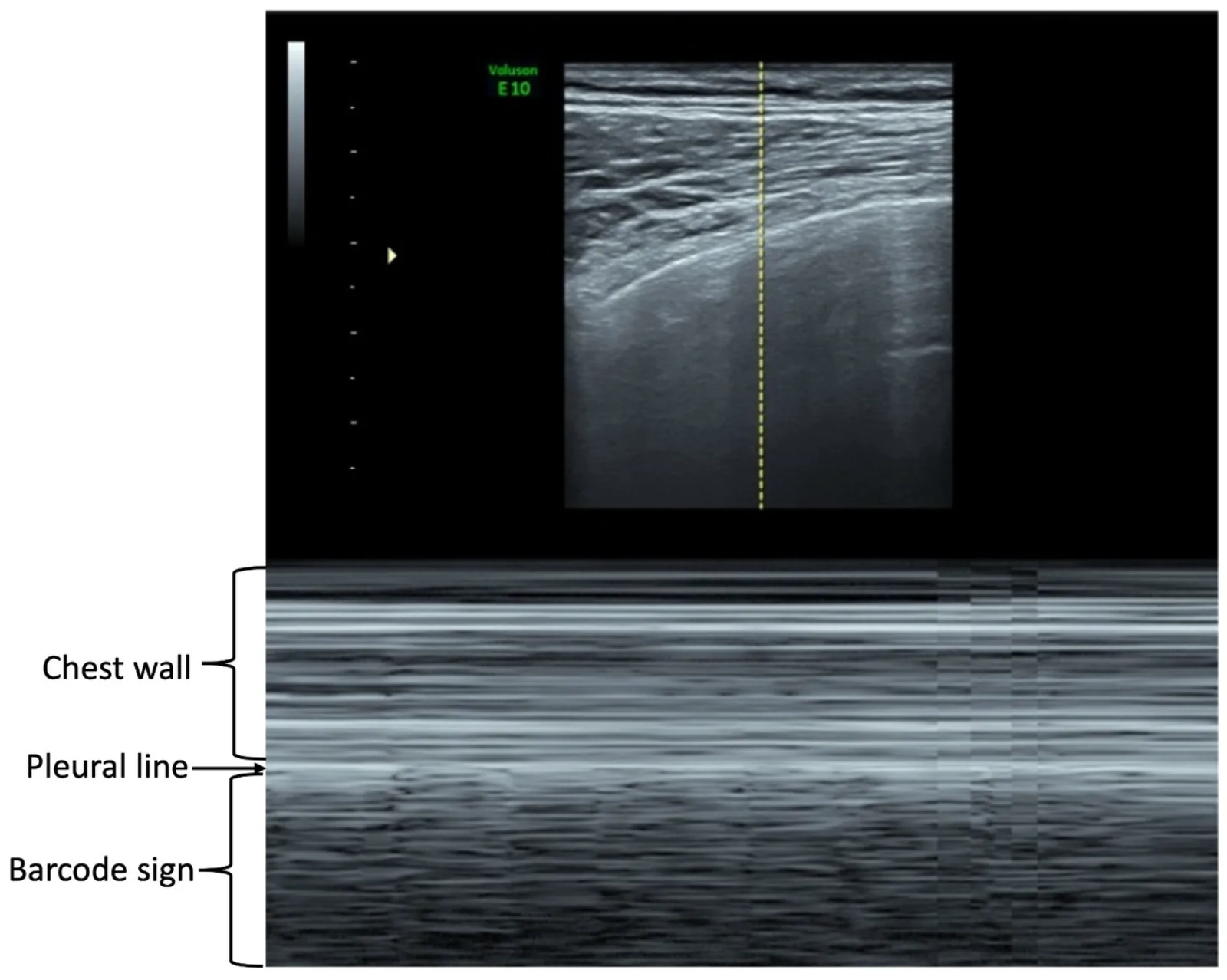
Right midaxillary M-mode transthoracic ultrasonography scan showing the absence of pleural-line motion (“barcode sign”) in a patient with severe asthma without pneumothorax. The yellow arrowhead indicates the focal zone, and the yellow dashed line indicates the sampling line used for M-mode examination.

Pleural sliding was defined as real-time movement of the hyperechoic pleural line during respiration and was evaluated with M-mode ultrasonography; a “barcode sign” on M-mode was taken to indicate absent pleural sliding (Figure 1).

**Figure 2:**
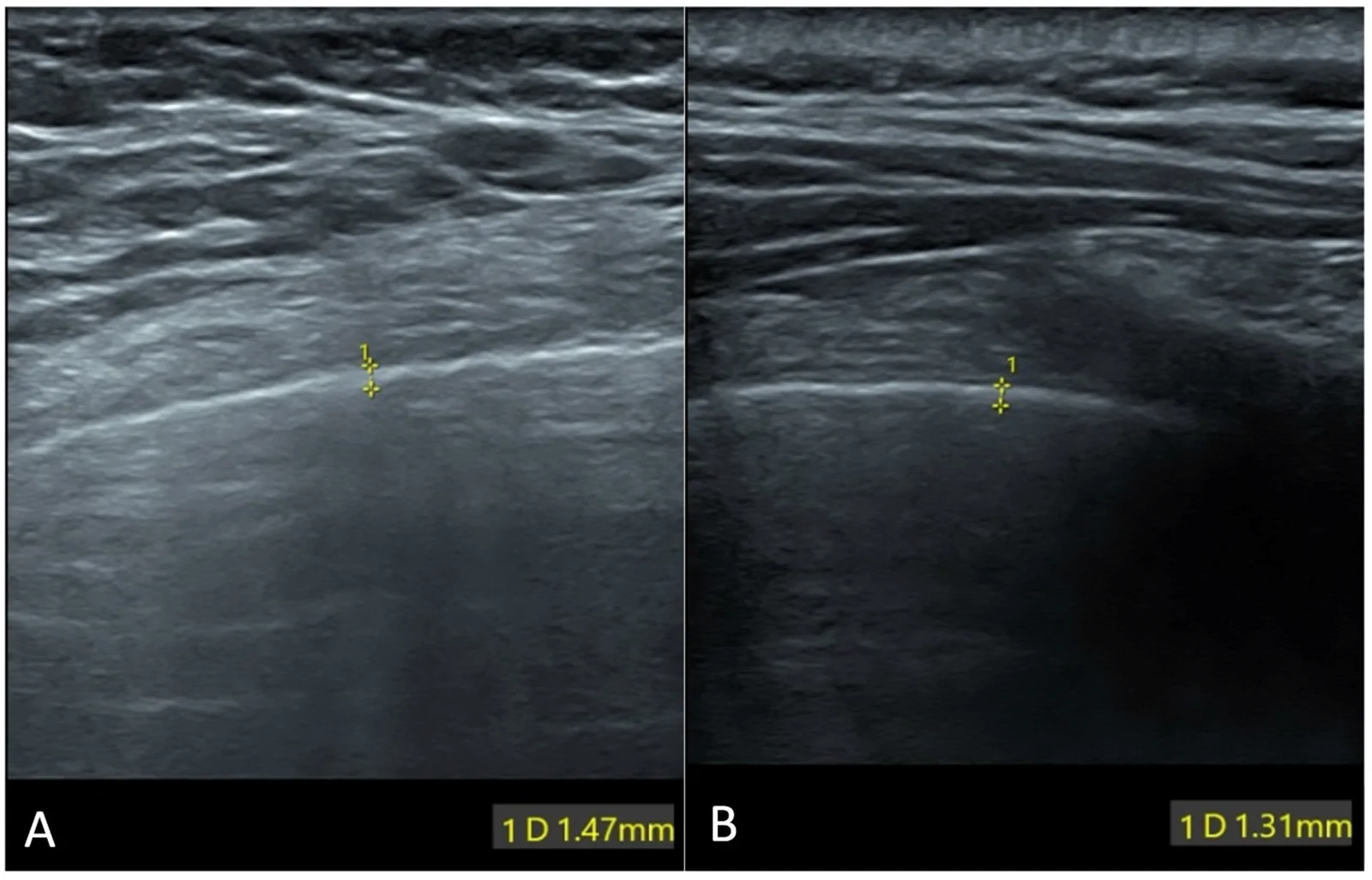
Transthoracic ultrasonography images of the left midclavicular zone before and after treatment in the same patient. (**A**) Baseline B-mode scan showing an irregular pleural line. (**B**) Week-16 B-mode scan at the same location, showing reduced pleural-line thickness and a regular pleural line. The yellow caliper markers indicate the boundaries used for pleural-line thickness measurement, and the numerical value displayed in the lower right corner indicates the measured thickness in millimeters.

Pleural-line thickness was measured in each of the 14 lung zones. Measurements were made perpendicular to the pleural line, with the caliper landmarks placed at the leading and trailing edges of the hyperechoic pleural line (Figure 2). Mean values across the specified zones were calculated separately for the right and left hemithorax. The change in pleural-line thickness (Δ) was calculated as post-treatment minus pre-treatment thickness (Δ = post-treatment − pre-treatment), so that a negative Δ indicates a reduction in pleural-line thickness and a positive Δ an increase.

Pleural-line morphology was classified in each of the 7 zones per hemithorax as regular or irregular (Figure 2). Each zone was scored 1 (regular) or 0 (irregular), and the scores were summed within each hemithorax to yield a pleural-line regularity score ranging from 0 (all zones irregular) to 7 (all zones regular). Right- and left-sided scores were analyzed separately; the combined bilateral score therefore ranges from 0 to 14. A patient in whom all 14 zones were regular was designated as having an entirely regular pleural line.

Subpleural nodules were defined as small hypoechoic lesions disrupting the continuity of the pleural line and were sought in each of the 14 zones in every patient at both examinations.

### 2.6. Statistical Analysis

The prespecified primary endpoint was the change in pleural-line thickness from baseline to week 16, calculated separately for the right and left hemithoraces as the mean of the seven lung zones within each hemithorax. Secondary outcomes were the pleural-line regularity score, pulmonary function, ACT and AQLQ scores, and the treatment-response classification. Secondary and subgroup analyses were regarded as exploratory and hypothesis-generating; their *p*-values were therefore interpreted cautiously and no formal adjustment for multiple comparisons was made.

The sample size was calculated a priori for the primary outcome, that is, the within-patient change in pleural-line thickness between baseline and week 16. In the absence of previous longitudinal TUS data in biologic-treated severe asthma, a moderate standardized effect size (Cohen’s dz = 0.5) was assumed. For a two-sided paired *t*-test with α = 0.05 and 80% power, a minimum of 34 evaluable patients was required; allowing for an anticipated 15% loss to follow-up, the planned sample size was approximately 40 patients. Consecutive eligible patients were recruited prospectively at a single center during the predefined study period. Recruitment ended when the predefined period closed, and 41 patients with complete paired assessments were included, exceeding the minimum required sample size. Although the sample size was calculated for a paired *t*-test, non-parametric testing was used for the primary endpoint where distributional assumptions were not met; given the high asymptotic relative efficiency of the Wilcoxon signed-rank test, this is expected to have had only a minor effect on statistical power.

Analyses were performed with IBM SPSS Statistics version 22.0 (IBM Corp., Armonk, NY, USA). Continuous variables are expressed as mean ± standard deviation or median (minimum–maximum) according to their distribution, and categorical variables as frequencies and percentages. Normality was assessed using the Kolmogorov–Smirnov and Shapiro–Wilk tests, and parametric or non-parametric tests were applied accordingly.

Pre- and post-treatment comparisons of continuous variables used the paired samples *t*-test for normally distributed data and the Wilcoxon signed-rank test for non-normally distributed data. Paired categorical variables were compared with the McNemar test, and other categorical comparisons with the chi-square test or Fisher’s exact test, as appropriate.

Complete responders and partial responders were compared with the Student’s *t*-test for normally distributed variables and the Mann–Whitney U test for non-normally distributed variables.

Associations between changes in TUS parameters and changes in clinical and functional variables were assessed with the Spearman rank correlation coefficient.

All tests were two-sided, and *p* < 0.05 was considered statistically significant for the primary endpoint.

## 3. Results

### 3.1. Patient Flow

**Figure 3:**
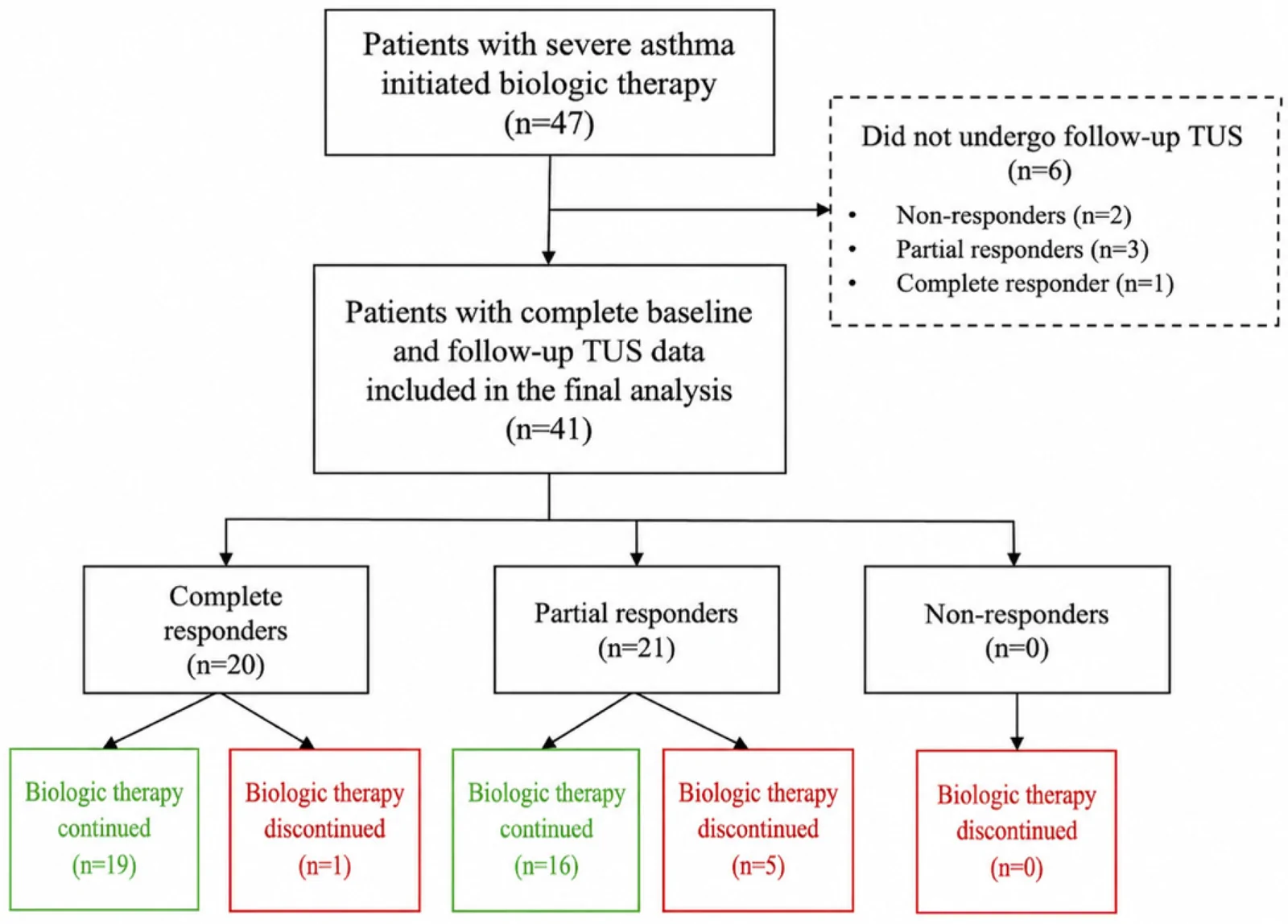
Flow chart of patient inclusion and follow-up.

A total of 47 patients with severe asthma started biologic therapy during the study period. Follow-up TUS was unavailable in six patients because the ultrasound examination was not performed after biologic therapy had been discontinued. Paired TUS analyses were therefore performed in the remaining 41 patients with complete baseline and week-16 ultrasound assessments (Figure 3).

Baseline demographic, clinical, functional, and TUS characteristics did not differ significantly between patients with and without follow-up TUS (all *p* > 0.05; Supplementary Table S1).

Among the 41 patients with paired TUS assessments, 20 (48.8%) were classified as complete responders and 21 (51.2%) as partial responders at week 16. No patient failed all evaluable domains, so the non-responder category was empty. Biologic therapy was discontinued in six patients overall (14.6%): five partial responders and one complete responder (Figure 3).

### 3.2. Baseline Characteristics of the Study Population

Of the 41 included patients, 33 (80.5%) were female and the mean age was 43.2 ± 13.2 years (Table 2).

The most common comorbidities were allergic rhinitis (51.2%), chronic rhinosinusitis (48.8%), and nasal polyps (34.1%). Skin prick testing demonstrated sensitization to house dust mites in 26 patients (63.4%), pollens in 15 (36.6%), cat dander in 10 (24.4%), cockroach in 5 (12.2%), and molds in 4 (9.8%).

All patients were receiving high-dose inhaled corticosteroid/long-acting β2-agonist therapy at baseline, and asthma therapy remained unchanged throughout the 16-week follow-up. Montelukast was the most frequently used add-on treatment (78.0%). Eighteen patients (43.9%) received omalizumab, 12 (29.3%) mepolizumab, and 11 (26.8%) benralizumab.

Complete and partial responders were comparable with respect to age, sex, body mass index, smoking status, asthma duration, and the distribution of biologic agents (all *p* > 0.05; Table 2). Among comorbidities, only hypertension differed between the groups, being more frequent in complete responders (*p* = 0.02), whereas montelukast use was more frequent in partial responders (*p* = 0.01). Complete responders had more severely impaired lung function at baseline: compared with partial responders, they had a lower FEV1 (75.2 ± 23.0% vs. 96.2 ± 22.4%, *p* = 0.01; 2.15 ± 1.1 L vs. 2.63 ± 0.6 L, *p* = 0.02), a lower FVC (86.0 ± 17.0% vs. 99.0 ± 16.2%, *p* = 0.02), a lower FEV1/FVC ratio (66.7 ± 12.3% vs. 75.2 ± 10.1%, *p* = 0.02), and a lower FEF25–75 (47.9 ± 27.5% vs. 74.4 ± 37.1%, *p* = 0.01). Complete responders also had a higher baseline blood eosinophil count (625 [20–1990] vs. 300 [0–1190] cells/µL, *p* = 0.01), whereas total serum IgE did not differ between the groups (*p* = 0.24). In contrast, no baseline transthoracic ultrasonography parameter differed according to subsequent treatment response.

The three treatment groups were not balanced at baseline: they differed significantly in age (*p* = 0.018), blood eosinophil count (*p* < 0.001), FEV1% predicted (*p* = 0.026), FVC% predicted (*p* = 0.036), and FEF25–75% predicted (*p* = 0.009), whereas baseline right and left pleural-line thickness were comparable across groups (*p* = 0.129 and *p* = 0.526, respectively).

### 3.3. Response to Biologic Therapy at 16 Weeks

Clinical, functional, and transthoracic ultrasound findings before and after 16 weeks of biologic therapy are summarized in Table 3. The mean ACT score increased from 13.2 ± 4.1 to 22.6 ± 2.9 and the mean AQLQ total score from 3.6 ± 1.2 to 4.9 ± 1.2 (both *p* < 0.001), with a significant improvement in all AQLQ domains (all *p* < 0.001). Lung function improved consistently: FEV1 rose from 86.0 ± 24.8% to 99.0 ± 20.9% predicted (*p* = 0.001) and FEF25–75 from 61.4 ± 35.0% to 81.2 ± 33.4% predicted (*p* < 0.001).

During the first 16 weeks of biologic therapy, 10 patients (24.4%) experienced an exacerbation requiring an emergency department visit and/or systemic corticosteroid treatment.

### 3.4. Ultrasonography Parameters During Treatment

Median pleural-line thickness decreased significantly after biologic therapy, from 1.27 (0.83–2.57) to 0.94 (0.61–1.34) mm in the right hemithorax and from 1.31 (0.89–2.20) to 0.97 (0.67–1.46) mm in the left hemithorax (both *p* < 0.001; Table 3). The pleural-line regularity score increased significantly on both sides (both *p* < 0.001; Table 3). Subpleural nodules were not observed in any patient at either examination.

Pleural-line thickness decreased significantly in all three biologic groups (all *p* ≤ 0.001), with no evidence of a differential treatment effect.

Pleural-line thinning was of similar magnitude in both response classes (Table 4). Within each class, the reduction was highly significant (all *p* < 0.001), but the magnitude of change did not differ between complete and partial responders on either side (mean Δ −0.33 vs. −0.42 mm on the right, *p* = 0.71; −0.36 vs. −0.37 mm on the left, *p* = 0.82). The same applied to the pleural-line regularity scores (*p* ≥ 0.41 for all comparisons).

### 3.5. Correlations Between Ultrasound Changes and Clinical Outcomes

Changes in right and left pleural-line thickness were moderately correlated with each other (r = 0.427, *p* = 0.01), indicating a consistent bilateral reduction in pleural-line thickness after biologic therapy. Spearman correlation analysis showed no significant association between changes in thoracic ultrasound parameters (Δ right and left pleural-line regularity score, Δ right and left pleural-line thickness) and changes in clinical outcomes (ΔACT, ΔAQLQ, ΔFEV1, ΔFEF; Table 5).

Improvement in ACT was moderately correlated with improvement in AQLQ (r = 0.551, *p* < 0.001), and changes in FEV1 were also positively correlated with changes in AQLQ (r = 0.437, *p* = 0.004).

## 4. Discussion

In this prospective study, transthoracic ultrasound parameters improved significantly after 16 weeks of biologic therapy in patients with severe asthma. The principal findings were a significant reduction in pleural-line thickness and an improvement in the pleural-line regularity score. Notably, although approximately half of the patients achieved a complete clinical response, the pleural ultrasound parameters improved irrespective of the response category. Improvement was seen with all three biologic agents, and no significant difference between agents remained after accounting for baseline characteristics. Consistent with the established clinical benefits of biologic therapy, pulmonary function also improved significantly, for both FEV1 and FEF25–75. To our knowledge, this is the first prospective study to describe longitudinal changes in pleural ultrasound parameters during biologic therapy in severe asthma.

Biologic therapy was associated with a significant reduction in pleural-line thickness on transthoracic ultrasonography, indicating that TUS can detect treatment-related change in the subpleural lung. Previous cross-sectional work has linked pleural abnormalities to peripheral airway disease: Scioscia et al. showed that pleural-line thickening and a lower pleural-line regularity score were strongly associated with distal airway inflammation and air trapping, with high agreement with HRCT findings [5], and Del Colle et al. and Martins et al. reported that abnormalities of pleural sliding and pleural-line morphology reflect bronchospasm and hyperinflation [13,14]. Beyond ultrasonography, evidence from CT, MRI, oscillometry, and endobronchial biopsy indicates that biologic agents targeting type 2 inflammation reduce airway wall thickening, mucus plug burden, ventilation defects, and other features of airway remodeling [15,16,17]. The histological substrate of the pleural line and the mechanisms underlying its thickening are not fully defined, and may involve subpleural interstitial change, air trapping at the lung–pleura interface, or local inflammation. Nevertheless, the longitudinal findings from this study extend this literature by showing that these ultrasound signals are dynamic and improve during biologic therapy. The reduction was modest in absolute terms (median Δ −0.33 mm on the right and −0.34 mm on the left) but corresponded to a relative decrease of approximately 26% from baseline on both sides. No minimal clinically important difference, normal reference range, or smallest detectable change has yet been established for pleural-line thickness in adults with asthma or other airway disease, and the available data are indirect. In neonates with and without acute respiratory failure, pleural-line thickness measured with high-frequency ultrasound showed excellent inter-rater agreement (ICC 0.95, 95% CI 0.94–0.96) with a coefficient of variation of 2.8% and 2.5% for end-inspiratory and end-expiratory measurements, respectively, together with a small but significant respiratory-phase dependence [18]. In our study, the observed reduction in pleural-line thickness was considerably larger than the coefficient of variation reported for standardized measurements [18], but this comparison is indirect and does not substitute for a reproducibility study in the present population. These findings should therefore be interpreted as demonstrating measurable group-level change associated with biologic therapy, rather than as defining a threshold for decision-making in an individual patient. Studies of measurement reproducibility and of clinically meaningful change, ideally anchored to established markers of small-airway disease such as HRCT or oscillometry, will be essential to determine the clinical utility of serial TUS. Combined with its practical advantages over conventional imaging—the absence of ionizing radiation, bedside applicability, low cost, and suitability for repeated longitudinal assessment—TUS may nonetheless offer a simple, non-invasive means of monitoring peripheral structural change during treatment.

A central and somewhat unexpected observation was that pleural-line thinning was dissociated from conventional measures of clinical response. The reduction did not differ between complete and partial responders, the pleural-line regularity score improved similarly in both groups, and changes in pleural-line thickness did not correlate with changes in ACT, AQLQ, FEV1, or FEF25–75, even though all of these improved significantly. FEF25–75 is frequently used as a spirometric indicator of small-airway function, and pleural abnormalities have been proposed to reflect the same compartment [5]. However, FEF25–75 shows substantial between- and within-subject variability and is mathematically dependent on FVC and on the volume over which it is measured; it should therefore be regarded as an exploratory physiological marker rather than as a specific measure of small-airway caliber. Despite a significant improvement in FEF25–75, its change showed essentially no correlation with pleural-line thinning. This suggests that pleural-line thickness is not simply a mechanical surrogate for airflow through the small airways, but may instead capture a structural or inflammatory dimension of the subpleural lung that follows its own trajectory. This dissociation is unlikely to be a chance finding, because the bilateral reduction was internally consistent—right- and left-sided changes correlated with each other—which supports a reproducible biological phenomenon rather than random measurement variability. One possible explanation is that the pleural signal reflects a distinct pathophysiological compartment, involving subpleural tissue and peripheral airway remodeling, which is only partly represented by conventional spirometric and clinical outcomes. In addition, the outcomes evaluated here are not purely structural. Symptoms, quality of life, and even spirometric performance are influenced not only by airway caliber and remodeling but also by neural regulation of breathing, including afferent airway signaling, central ventilatory control, and peripheral chemoreflex sensitivity. Alterations in these mechanisms have been described across pulmonary diseases and may influence breathlessness and ventilatory responses independently of structural airway change [19]. Structural improvement at the lung–pleura interface would therefore not necessarily be expected to parallel improvement in symptoms or spirometric indices. Differences in the timing of recovery may provide a further explanation. Structural remodeling, pulmonary mechanics and neural control are unlikely to recover synchronously after pulmonary injury or during biologic treatment. Experimental studies have shown that physiological responses, including peripheral chemoreflex sensitivity, evolve dynamically during recovery rather than following a single trajectory [20]. Although these observations derive from experimental lung injury rather than severe asthma, they support the broader concept that different components of respiratory recovery may follow distinct temporal patterns. Assessment at a single 16-week time point may therefore have captured structural, functional, and clinical recovery at different stages of their evolution, reducing the likelihood of detecting cross-sectional correlations. Serial TUS examinations over a longer follow-up will be important to determine whether pleural-line changes continue to evolve after clinical improvement and to clarify their temporal relationship with conventional markers of treatment response. This interpretation is consistent with the growing recognition of small-airway disease as a relatively independent treatable trait in severe asthma [4,21]. TUS-derived pleural-line assessment should, accordingly, be regarded as a complementary imaging biomarker that provides information on peripheral structural remodeling, rather than as a stand-alone measure of treatment response. Such complementary information may become particularly relevant to emerging concepts of asthma remission, which increasingly emphasize resolution of underlying inflammation and structural remodeling in addition to symptom control and lung function. It should nevertheless be noted that the absence of correlation may also partly reflect the limited sample size, the relatively narrow range of clinical improvement observed after treatment, and the small absolute magnitude of the pleural changes, which approaches the resolution of the measurement.

Pleural-line thinning was observed with all three biologic agents. Although the study was not designed to compare individual agents, the absence of an agent-specific pattern is noteworthy, because anti–IL-5 therapy—particularly benralizumab—has been reported to exert the greatest effect on physiological markers of small-airway dysfunction, including lung hyperinflation, air trapping, and FEF25–75 [22]. The lack of a corresponding gradient in pleural-line thickness again suggests that this parameter is not simply a surrogate for small-airway physiology, and that it may instead capture subpleural structural or inflammatory change common to several type 2–targeting mechanisms [23]. This interpretation remains hypothesis-generating and requires confirmation in adequately powered, mechanistically anchored studies.

The magnitude of clinical benefit observed here—a 9.4-point gain in ACT, a 1.3-point improvement in AQLQ, and a 13-point rise in FEV1% predicted and a nearly 20-point rise in FEF25–75% predicted—is consistent with, and at the upper end of, that reported in real-world biologic cohorts. In the International Severe Asthma Registry, biologics produced marked improvements in control and exacerbation rates, with super-response in a substantial minority of patients [24], and the three-year SANI single-center experience reported sustained gains in control and lung function with anti–IL-5 therapy [25]. The 48.8% complete-response rate in the present cohort falls within the range reported when composite, guideline-based definitions are applied [12] and reinforces the point made by Mailhot-Larouche et al. and Scelo et al. that the definition of response strongly shapes the observed rate [3,12]. The dissociation between robust symptomatic improvement and more modest spirometric change echoes the well-described gap between patient-reported and physiological responses to biologics [3] and motivates the search for objective, tissue-level markers such as TUS. If validated, serial TUS could provide a radiation-free means of tracking tissue-level response—for example, in patients whose FEV1 and FEF25–75 are already near-normal but in whom ongoing remodeling is suspected, or as an adjunct when assessing progress toward remission.

### 4.1. Limitations of the Study

This study has several limitations. First, it was a single-arm study without an untreated or placebo control group. Although the pre-imaging bronchodilator washout and the standardized image acquisition reduce short-term confounding, time-related or non-biologic contributions cannot be excluded, and the findings should therefore be interpreted as associations rather than as proof of an effect specific to biologic therapy. Second, follow-up was limited to 16 weeks, so the durability and prognostic significance of the pleural changes remain unknown. Third, the pleural-line regularity score is a semi-quantitative measure that has not yet been externally validated. Fourth, intra- and inter-observer reproducibility was not formally assessed; although all examinations were performed by a single experienced radiologist using the same ultrasound system, identical machine settings and a standardized protocol at both visits, the reproducibility of pleural-line thickness measurements remains to be established. Fifth, no concurrent reference standard (HRCT, FeNO, oscillometry or histology) was obtained; in particular, FEF25–75 was the only available measure of small-airway function, whereas impulse oscillometry or HRCT air-trapping indices would provide a more direct assessment, so the mechanistic relationship between pleural-line change and peripheral airway remodeling could not be established directly. Finally, in the absence of a formal reproducibility study, the smallest detectable change for pleural-line thickness could not be determined; together with the lack of an established minimal clinically important difference and of normal reference values, this means that the clinical interpretation of changes in individual patients remains uncertain despite the robust group-level improvement observed.

### 4.2. Recommendations for Future Research

Several priorities follow from these findings. First, controlled studies, including an untreated or standard-of-care comparator group, are required to establish whether the observed pleural changes are attributable to biologic therapy rather than to time or to concurrent treatment optimization of treatment. Second, reproducibility studies reporting intra- and inter-observer agreement and the smallest detectable change are needed before pleural-line thickness can be interpreted at the level of the individual patient. Third, concurrent assessment against established structural and inflammatory reference standards—HRCT air-trapping indices, impulse oscillometry and FeNO—would clarify what the pleural signal represents biologically. Fourth, longer follow-up with repeated measurements would establish the durability of the observed changes and determine whether an early pleural response predicts subsequent exacerbation risk. Finally, multicenter studies with larger and more balanced treatment groups would be needed to determine whether the response differs between individual biologic agents, a question this study was neither designed nor powered to address.

## 5. Conclusions

In patients with severe asthma, biologic therapy was associated with a measurable reduction in pleural-line thickness and improved pleural-line regularity score on transthoracic ultrasound. These pleural changes accompanied improvements in symptoms, quality of life, and lung function but did not correlate with them, suggesting that TUS captures a complementary, tissue-level dimension of the response to treatment.

## Figures and Tables

**Table 1:** Criteria for classifying treatment response at week 16 after initiation of biologic therapy.

Response Category	Definition
Complete responder	Met the criteria in all three of the following domains: Asthma control: ≥3-point improvement in ACT and/or ACT ≥20. Lung function: ≥100 mL and/or ≥10% improvement in FEV1. Exacerbations: no exacerbation and no systemic corticosteroid course during the 16-week period.
Partial responder	Met criteria in one or two of the three domains.
Non-responder	Met the criteria in none of the three domains.

ACT, asthma control test; FEV1, forced expiratory volume in 1 s.

**Table 2:** Baseline characteristics according to treatment response to biologic therapy.

	Total (*n* = 41)	Complete Responders (*n* = 20)	Partial Responders (*n* = 21)	*p*-Value
Age, years, mean ± SD	43.2 ± 13.2	47.2 ± 14.7	39.5 ± 10.5	0.06
Gender, Female, *n* (%)	33 (80.5)	15 (75)	18 (85.7)	0.45
BMI, kg/m^2^, median (min–max)	29.2 (20.9–44.7)	26.5 (20.9–37.6)	30.8 (21–44.7)	0.12
Never smoker, *n* (%)	30 (73.2)	14 (70)	16 (76.2)	0.92
Comorbidities, *n* (%)				
Allergic rhinitis	21 (51.2)	9 (45)	12 (57.1)	0.64
Chronic sinusitis	20 (48.8)	10 (50)	10 (47.6)	1.00
Nasal polyps	14 (34.1)	7 (35)	7 (33.3)	1.00
Food allergy	3 (7.3)	0 (0)	3 (14.3)	0.23
Drug allergy	2 (4.9)	0 (0)	2 (9.5)	0.49
Anxiety/Depression	4 (9.8)	2 (10)	2 (9.5)	1.00
Hypertension	8 (19.5)	7 (35)	1 (4.8)	**0.02**
Diabetes mellitus	5 (12.2)	3 (15)	2 (9.5)	0.66
Additional medications, *n* (%)				
Montelukast	32 (78)	12 (60)	20 (95.2)	**0.01**
LAMA	29 (70.7)	17 (85)	12 (57.1)	0.11
Biologic therapy, *n* (%)				
Omalizumab	18 (43.9)	6 (30)	12 (57.1)	0.15
Mepolizumab	12 (29.3)	8 (40)	4 (19)	0.26
Benralizumab	11 (26.8)	6 (30)	5 (23.8)	0.92
Asthma duration, years, median (min–max)	10 (1–50)	10 (1–50)	9 (1–35)	0.83
Initial asthma condition, median (min–max)				
ACT score, mean ± SD	13.2 ± 4.1	11.95 ± 4.5	14.5 ± 3.4	0.05
Emergency visits in previous year	3 (0–48)	3 (0–24)	4 (0–48)	0.35
OCS courses in previous year	2 (0–12)	2.5 (0–12)	2 (0–6)	0.19
Hospitalizations in previous year	0 (0–3)	0 (0–3)	0 (0–2)	0.58
Spirometry, mean ± SD				
FEV1/FVC, %	71.1 ± 11.9	66.7 ± 12.3	75.2 ± 10.1	**0.02**
FEV1, L	2.39 ± 0.9	2.15 ± 1.1	2.63 ± 0.6	**0.02**
FEV1, %	85.98 ± 24.8	75.2 ± 22.95	96.2 ± 22.4	**0.01**
FVC, L	3.33 ± 1.1	3.17 ± 1.4	3.47 ± 0.6	0.38
FVC, %	92.6 ± 17.6	86 ± 17	98.95 ± 16.2	**0.02**
FEF25–75, L	1.92 ± 1.2	1.45 ± 1.1	2.37 ± 1.2	**0.01**
FEF25–75, %	61.4 ± 35	47.9 ± 27.5	74.4 ± 37.1	**0.01**
Laboratory findings, median (min–max)				
Total serum IgE, kU/L	192.4 (24.3–2243.2)	252.1 (35.6–2243.2)	153.9 (24.3–1303.8)	0.24
Blood eosinophil, cells/µL	400 (0–1990)	625 (20–1990)	300 (0–1190)	**0.01**
Baseline TUS findings, median (min–max)				
Right pleural-line thickness, mm	1.27 (0.83–2.57)	1.21 (0.99–1.96)	1.43 (0.83–2.57)	0.33
Left pleural-line thickness, mm	1.31 (0.89–2.20)	1.28 (0.94–1.94)	1.34 (0.89–2.20)	0.68
Right pleural-line regularity score	4 (3–7)	4 (3–7)	4 (3–7)	0.34
Left pleural-line regularity score	5 (2–7)	5 (2–7)	4 (2–7)	0.31
Pleural sliding, *n* (%)	32 (78)	17 (85)	15 (71.4)	0.45
Regular pleural line, *n* (%)	5 (12.2)	4 (20)	1 (4.8)	0.18

Significant *p*-values are shown in bold. Values are presented as mean ± SD, median (min–max), or *n* (%), as appropriate. *p*-values were calculated using Fisher’s exact test when expected cell counts were <5. BMI, body mass index; LAMA, long-acting antimuscarinic; OCS, oral corticosteroids; ACT, asthma control test; FEV1, forced expiratory volume in 1 s; FVC, forced vital capacity; FEF25–75, forced expiratory flow between 25% and 75% of vital capacity; SD, standard deviation; TUS, transthoracic ultrasound.

**Table 3:** Clinical, functional, and ultrasonography parameters at baseline and week 16 (whole cohort, *n* = 41).

	Baseline	Week 16	*p*-Value
ACT score, mean ± SD	13.2 ± 4.1	22.6 ± 2.9	**<0.001**
AQLQ total score, mean ± SD	3.6 ± 1.2	4.9 ± 1.2	**<0.001**
AQLQ score, mean ± SD			
Symptom	3.4 ± 1.3	4.8 ± 1.3	**<0.001**
Activity limitation	3.6 ± 1.1	4.7 ± 1.3	**<0.001**
Emotional function	3.8 ± 1.5	4.97 ± 1.3	**<0.001**
Environmental stimuli	3.9 ± 1.8	5.3 ± 1.3	**<0.001**
Pulmonary function test, mean ± SD			
FEV1, % predicted	85.98 ± 24.8	99.0 ± 20.9	**0.001**
FEV1, L	2.39 ± 0.9	2.77 ± 0.9	**<0.001**
FVC, % predicted	92.6 ± 17.6	99 ± 16	**0.01**
FVC, L	3.33 ± 1.1	3.59 ± 1	**0.004**
FEV1/FVC (%)	71.1 ± 11.9	76.9 ± 9.4	**<0.001**
FEF25–75, % predicted	61.4 ± 35	81.2 ± 33.4	**<0.001**
FEF25–75, L	1.92 ± 1.2	2.54 ± 1.2	**<0.001**
Pleural sliding, *n* (%)	32 (78)	37 (90.2)	0.18
Pleural-line thickness, mm, median (min–max)			
Right	1.27 (0.83–2.57)	0.94 (0.61–1.34)	**<0.001**
Left	1.31 (0.89–2.20)	0.97 (0.67–1.46)	**<0.001**
Regular pleural line, *n* (%)	5 (12.2)	10 (24.4)	0.18
Pleural-line regularity score, median (min–max)			
Right	4 (3–7)	6 (3–7)	**<0.001**
Left	5 (2–7)	6 (3–7)	**<0.001**
Subpleural nodules, *n* (%)	0 (0)	0 (0)	-

Significant *p*-values are shown in bold. ACT, Asthma Control Test; AQLQ, Asthma Quality of Life Questionnaire; FEV1, forced expiratory volume in 1 s; FVC, forced vital capacity; FEF25–75, forced expiratory flow between 25% and 75% of vital capacity; SD, standard deviation.

**Table 4:** Changes in ultrasonography parameters according to treatment-response status.

	Complete Responders (*n* = 20)			Partial Responders (*n* = 21)			
TUS Findings, Median (Min–Max)	Baseline	Week 16	*p*-Value	Baseline	Week 16	*p*-Value	Between- Group *p* *
Right pleural-line thickness, mm	1.21 (0.99–1.96)	0.99 (0.70–1.30)	<0.001	1.43 (0.83–2.57)	0.93 (0.61–1.34)	<0.001	0.71
Left pleural-line thickness, mm	1.28 (0.94–1.94)	0.93 (0.77–1.27)	<0.001	1.34 (0.89–2.20)	0.97 (0.67–1.46)	<0.001	0.82
Right pleural-line regularity score	4 (3–7)	6 (4–7)	0.001	4 (3–7)	6 (3–7)	<0.001	0.45
Left pleural-line regularity score	5 (2–7)	6 (5–7)	0.001	4 (2–7)	6 (3–7)	<0.001	0.41

* Between-group *p*-values compare the magnitude of change (Δ) between complete responders and partial responders. TUS, transthoracic ultrasound.

**Table 5:** Correlations between the change in pleural-line thickness and changes in functional and clinical variables (all Δ = post − pre).

	Δ Right Pleural-Line Thickness		Δ Left Pleural-Line Thickness	
Variable (Δ = Week 16 − Baseline)	*r*	*p*	*r*	*p*
Δ FEF25–75 (%)	0.038	0.814	0.116	0.471
Δ FEF25–75 (L)	0.046	0.776	0.132	0.410
Δ FEV1 (%)	0.189	0.236	0.195	0.223
Δ FEV1 (L)	0.164	0.307	0.170	0.288
Δ ACT score	0.181	0.259	0.104	0.518
Δ AQLQ score	0.010	0.949	−0.109	0.498

Spearman rank correlation. All Δ values are week 16 − baseline (post − pre): a negative Δ pleural-line thickness denotes thinning (improvement). ACT, Asthma Control Test; AQLQ, Asthma Quality of Life Questionnaire; FEF25–75, forced expiratory flow 25–75%; FEV1, forced expiratory volume in 1 s.

## Data Availability

The datasets generated and/or analyzed during this study are not publicly available because they contain information that could compromise participant privacy and because of ethical restrictions. The data are available from the corresponding author upon reasonable request, subject to approval by the Karadeniz Technical University Ethics Committee and applicable institutional regulations.

## Supplementary Materials

The following supporting information can be downloaded at: https://www.mdpi.com/article/10.3390/jcm15166346/s1, Table S1: Baseline demographic, clinical, functional and thoracic ultrasonography characteristics of patients included in versus excluded from the paired analysis.
