# Supplementary material for: Biologic Therapy Is Associated with Reduced Pleural-Line Thickness in Severe Asthma: A Prospective Transthoracic Ultrasound Study
Source: J Clin Med. 2026 Aug 17;15(16):6346. doi: 10.3390/jcm15166346 (PMC13513467; doi:10.3390/jcm15166346)
Supplement: Supplementary file 1 [file jcm-15-06346-s001.zip › jcm-4463596-supplementary.pdf]

Table S1. Baseline demographic, clinical, functional and thoracic ultrasonography characteristics of patients included in versus excluded from the paired analysis.

Baseline variable	Included in paired analysis (n = 41)	Excluded, no follow-up TUS (n = 6)	p*
Age, years	43.0 (22.0–77.0)	55.5 (23.0–74.0)	0.138
Female sex, n (%)	33 (80.5)	4 (66.7)	0.594
BMI, kg/m²	29.2 (20.9–44.7)	35.4 (21.5–48.6)	0.107
Current smoker, n (%)	10 (24.4)	2 (33.3)	0.637
Asthma duration, years	10.0 (1.0–50.0)	12.5 (3.0–40.0)	0.543
Allergic rhinitis, n (%)	21 (51.2)	3 (50.0)	1.000
Chronic sinusitis, n (%)	20 (48.8)	2 (33.3)	0.670
Nasal polyps, n (%)	14 (34.1)	0 (0.0)	0.159
Food allergy, n (%)	3 (7.3)	0 (0.0)	1.000
Drug allergy, n (%)	2 (4.9)	0 (0.0)	1.000
Anxiety/depression, n (%)	4 (9.8)	1 (16.7)	0.511
Hypertension, n (%)	8 (19.5)	1 (16.7)	1.000
Diabetes mellitus, n (%)	5 (12.2)	0 (0.0)	1.000
Montelukast use, n (%)	32 (78.0)	3 (50.0)	0.164
LAMA use, n (%)	29 (70.7)	5 (83.3)	1.000
Blood eosinophils, /µL	400.0 (0.0–1990.0)	345.0 (140.0–420.0)	0.513
Total serum IgE, IU/mL	192.4 (24.3–2243.2)	404.2 (40.8–825.0)	0.544
FEV1, % predicted	86.0 (37.0–124.0)	80.0 (58.0–106.0)	0.702
FEV1, L	2.2 (0.8–4.8)	1.7 (1.5–4.0)	0.208
FVC, % predicted	91.0 (57.0–127.0)	80.5 (62.0–95.0)	0.118
FEV1/FVC, %	72.0 (41.0–94.0)	76.0 (65.0–94.0)	0.339
FEF25–75, % predicted	62.0 (8.0–164.0)	63.0 (34.0–125.0)	0.463
ACT score	13.0 (5.0–20.0)	11.5 (7.0–16.0)	0.423
AQLQ total score	3.3 (1.4–6.3)	2.7 (2.3–3.7)	0.111
Right pleural-line thickness, mm	1.3 (0.8–2.6)	1.3 (1.3–1.7)	0.389
Left pleural-line thickness, mm	1.3 (0.9–2.2)	1.4 (1.1–1.7)	0.483
Absent pleural sliding, n (%)	9 (22.0)	2 (33.3)	0.614
Pleural-line regularity, n (%)	5 (12.2)	0 (0.0)	1.000
Right pleural-line regularity score	4.0 (3.0–7.0)	4.5 (3.0–7.0)	0.779
Left pleural-line regularity score	5.0 (2.0–7.0)	5.0 (4.0–6.0)	0.661
Omalizumab, n (%)	18 (43.9)	2 (33.3)	1.000
Mepolizumab, n (%)	12 (29.3)	1 (16.7)	1.000
Benralizumab, n (%)	11 (26.8)	3 (50.0)	0.344

Values are median (minimum–maximum) for continuous variables and n (%) for categorical variables. *Mann–Whitney U test for continuous variables and Fisher's exact test for categorical variables; no comparison reached statistical significance (all $p > 0.05$). Because only six patients were excluded, these comparisons had limited statistical power to detect between-group differences. ACT, Asthma Control Test; AQLQ, Asthma Quality of Life Questionnaire; BMI, body mass index; FEF25–75, forced expiratory flow 25–75%; FEV1, forced expiratory volume in 1 s; FVC, forced vital capacity; IgE, immunoglobulin E; LAMA, long-acting muscarinic antagonist; TUS, transthoracic ultrasonography.
